# Cryptococcus spp isolated from dust microhabitat in Brazilian libraries

**DOI:** 10.1186/1745-6673-7-11

**Published:** 2012-06-08

**Authors:** Diniz P Leite, Janaina V R S Amadio, Evelin R Martins, Sara A A Simões, Ana Caroline A Yamamoto, Fábio A Leal-Santos, Doracilde T Takahara, Rosane C Hahn

**Affiliations:** 1 Medicine Faculty, Mycology laboratory, Federal University of Mato Grosso, Fernando Correa da Costa Avenue, 2367, Boa Esperança - Cuiabá/MT, Brazil

**Keywords:** *Cryptococcus* spp, Dust, Libraries, Air, Habitat

## Abstract

**Background:**

The *Cryptococcus* spp is currently composed of encapsulated yeasts of cosmopolitan distribution, including the etiological agents of cryptococcosis. The fungus are found mainly in substrates of animal and plant origin. Human infection occurs through inhalation of spores present in the environment.

**Methods:**

Eighty-four swab collections were performed on dust found on books in three libraries in the city of Cuiabá, state of Mato Grosso, Brazil. The material was seeded in Sabouraud agar and then observed for characteristics compatible with colonies with a creamy to mucous aspect; the material was then isolated in birdseed (Niger) agar and cultivated at a temperature of 37°C for 5 to 7 days. Identification of isolated colonies was performed by microscopic observation in fresh preparations dyed with India ink, additional tests performed on CGB (L-canavanine glycine bromothymol blue), urea broth, and carbohydrate assimilation tests (auxanogram).

**Results:**

Of the 84 samples collected from book dust, 18 (21.4%) were positive for *Cryptococcus* spp totalizing 41 UFC’s. The most frequently isolated species was *C. gattii* 15 (36.6%); followed by *C. terreus,* 12 (29.3%); *C. luteolus* 4 (9.8%); *C. neoformans*, and *C. uniguttulatus* 3 (7.3%), and *C. albidus* and *C. humiculus* with 2 (4.6%) of the isolates.

**Conclusion:**

The high biodiversity of the yeasts of the *Cryptococcus* genus, isolated from different environmental sources in urban areas of Brazil suggests the possibility of individuals whose immune systems have been compromised or even healthy individuals coming into sources of fungal propagules on a daily bases throughout their lives. This study demonstrates the acquisition possible of cryptococcosis infection from dust in libraries.

## Background

Since moving to *Cryptococcus* spp was first isolated, over 113 years ago, many studies have been conducted on the subject. However, many clinical-epidemiological and ecological aspects are still unknown, especially in Brazil. Regarding the epidemiology of the agent, there is a wide variety of species distributed in different countries and in different regions of the same country [1], and there are also many areas which are yet to be researched in regard to the interaction of the agent with its human host [2].

**Figure 1:**
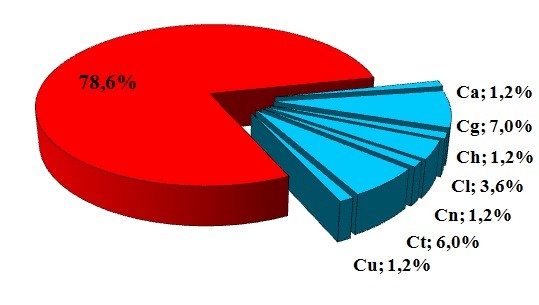
**Presence of *Cryptococcus *spp in 84 samples taken from the dust substrate of three libraries of Cuiabá, MT.**

Yeasts of the *Cryptococcus* genus have a peculiar type of geographic distribution, considering their species. A high incidence of *Cryptococcus neoformans* is found in European countries and in North America; whereas *Cryptococcus gattii * is found predominantly in tropical and subtropical regions. This peculiar geographic distribution is a significant factor in the study of the natural habitat of *Cryptococcus*, as a possible source of contagion for susceptible individuals [3].

Five serotypes are currently recognized for this fungus (A, B, C, D, and AD). The distinction between different serotypes is based on the immunologic reaction to the antiserum produced against different polysaccharide compositions that constitute the yeast capsule. The AD serotype was classified as a diploid hybrid of A and D serotypes [1]. Recent DNA polymorphism studies using AFLP (*amplified fragment length polymorphism*). Demonstrated that the genetic differences between varieties were enough to classify them as two distinct species: *Cryptococcus neoformans* and *Cryptococcus gattii *[4-7].

The distribution of the yeasts of the genus *Cryptococcus* in nature is very large and is associated especially to decomposing vegetables, fruits, and bird droppings, particularly pigeon droppings [8]. The species *C. neoformans* is commonly isolated from bird excreta, urban pigeons (*Columba livia*) being considered the main source of this species [9,10]; *C. gattii* has been isolated mainly from samples of eucalyptus (*Eucalyptus camaldulensis, E. tereticornis*, and *E. gomphocephala)*[11-15] and other trees in Brazil [16-19].

In many situations, reports of cryptococcosis have been associated to pigeon droppings as the source of the infection. However, epidemiological analyses showed that patients who are in contact with pigeons are subjected to a high risk of infection [9,10]. The main problem is that *C. neoformans* remains viable for many years in dry pigeon droppings, which become a reservoir for infectious particles accumulated by alveolar deposition. These yeasts are currently present in urban areas and associated with urban birds and their habitats, which are adapted to buildings, such as pigeons, and birds that live in parks, commercial establishments, and homes, such as parakeets and canaries [20-22].

The *Cryptococcus* genus is composed of approximately 34 species of yeast which reproduce asexually by budding and can be identified by: starch hydrolysis, assimilation of inositol, production of urease, non fermentation of sugars, and sensitivity to cycloheximide [23,24].

Studies to identify, isolate, and monitor the incidence of specific fungus species in different habitats still need to be conducted, since the literature reports urban environmental sources and plant substrates as the main environments harboring *Cryptococcus* spp, emphasizing sources associated to the exposure to pigeons and tree hollows in urban centers. In the study of cryptococcosis and its etiologic agents it is important to be aware of and extensively monitor reservoirs and sources of infection [8]. Therefore, these parameters guide epidemiological data for the implementation of prevention programs and effective therapies [25]. This study represents the first Brazilian report on the association between this fungus and dust found in libraries.

## Materials and methods

Eighty-four samples were collected inside three libraries (A, B, and C) and analyzed, in the city of Cuiabá, state of Mato Grosso, Brazil. The study was authorized by the people in charge of the libraries. The dust was collected with sterile swabs and placed in 20% sterile saline solution, and later transported to the mycology investigation laboratory of the Federal University of Mato Grosso for isolation and identification of the fungal microorganisms.

The colonies selected from the seeded plates (primary colonies) were re-isolated and diluted in 2.0 ml sterile water solution with chloramphenicol; they were later seeded on Sabouraud medium with chloramphenicol and incubated for 5 to 7 days at 37°C. After growth, the colonies were identified by morpho-physiological tests.

The macroscopic analysis of the colonies suggestive of *Cryptococcus* spp was conducted by observing the shiny, smooth aspect of the surface, with a creamy to mucous consistency, and a white to beige coloration. The micromorphology of the colonies was analyzed through microculture (Ridell technique). The isolates were submitted to a urease test and microscopic analysis with India ink to visualize the capsule [26].

The colonies were seeded in birdseed agar (Staib agar), which is recommended to verify phenoloxidase activity of the most studied pathogenic species nowadays, *Cryptococcus neoformans* and *Cryptococcus gattii *[9]. *C. neoformans* and *C. gattii* are the only yeasts of this kind capable of synthesizing melanin through the conversion of hydroxybenzoic substrates by phenoloxidase activity [27]. After passage through birdseed agar, the possible colonies which showed a coffee-brown coloration characteristic of *C. neoformans* and *C. gattii* were seeded in CGB medium (L-canavanine glycine bromothymol blue) for species identification [27].

For the biochemical tests, auxanogram technique was used, in which the assimilation of eleven carbon sources (dextrose, lactose, maltose, sucrose, inositol, galactose, cellobiose, dulcitol, melibiose, trehalose, and raffinose) and two nitrogen sources (peptone and potassium nitrate) [27] was used to differentiate, identify, and confirm at the species level.

## Results and discussion

Brazilian scientific literature on cryptococcosis has had a significant contribution in directing and explaining facts related to the agent of this disease. The habitat of yeasts of the genus *Cryptococcus*, especially *C. neoformans*, are found in the environment, principally in soil made up of decomposing plant material and bird and bat droppings found in both urban and rural areas in Brazil [1,9,10,20,22,28]. However, other studies have shed more light on the ecology of the cryptococcosis agent in Brazil, demonstrating that the yeast is associated not only with pigeons or *Eucalyptus* spp trees, but also with other tree species, as reported by Lazéra et al. [16-19] (and fellow authors), Fortes et al. [29] and Baltazar and Ribeiro [30].

In this study, the identification of seven different species of *Cryptococcus* yeasts, isolated from the dust found on books, indicates that dust is a possible biotope for isolating this type of microorganism (Figure 1). Forty-one colonies were isolated distributed through all three libraries and in two of the libraries, the presence of encapsulated yeast was detected in these environments. *Cryptococcus gattii * was the species most frequently identified, totaling 15 (36.6%) isolates; followed by *C. terreus* with 12 (29.3%) isolates and *C. luteolus* with 4 (9.8%) isolates. The other species detected were *C. neoformans* and *C. uniguttulatus* represented by 3 (7.3%) isolates each, and by *C. albidus* and *C. humiculus* with 2 (4.6%) isolates each, which presented the smallest percentiles (Table 1).

**Table 1:** **Presence of*Cryptococcus*spp in 84 samples taken from the dust substrate of three libraries of Cuiabá, MT**

	**Library/Relative frequency**							
ISOLATED SPECIES	B1	%	B2	%	B3	%	UFC	T%
*Cryptococcus albidus*	-	-	2	22.2	-	-	2	4.9
*Cryptococcus gattii*	12	37.5	3	33.3	-	-	15	36.6
*Cryptococcus humiculus*	2	6.3	-	-	-	-	2	4.9
*Cryptococcus luteolus*	4	12.5	-	-	-	-	4	9.8
*Cryptococcus neoformans*	3	9.4	-	-	-	-	3	7.3
*Cryptococcus terreus*	8	25.0	4	44.4	-	-	12	29.3
*Cryptococcus uniguttulatus*	3	9.4	-	-	-	-	3	7.3
TOTAL	32	100	9	100	-	-	41	100

Table 1. Frequency of colonies of isolates of yeasts species of the genus *Cryptococcus* from dust collected in three libraries in Cuiabá, MT, Brazil.

In Brazil, this encapsulated yeast has been isolated in several geographical regions: Bahia [20,31]; São Paulo [32,33]; Mato Grosso do Sul [9]; Goiás [34]; Rio de Janeiro [10,16,22] and Rio Grande do Sul [35-37]. In Mato Grosso, the first description of these microorganisms in HIV-positive patients was reported by Favalessa et al. [38]. The authors detected 37 C*. neoformans* and C*. gattii* isolates in distinct clinical materials from seropositive and seronegative patients.

The percentage (21.4%) of positive samples in this study (Chart 1) conducted in the city of Cuiabá is noteworthy compared with studies by other authors conducted in other Brazilian cities, when these involved bird droppings as substrates in the research. Soares et al. [39], in a study conducted in the city of Santos, obtained a frequency of 13.9% for pigeon excreta samples positive for *C. neoformans.* In the city of Goiânia, Kobayashi et al. [34] verified a frequency of 23.2%; while researching in church towers in Rio de Janeiro, Baroni et al. [10] observed that 37.8% of the samples collected showed the presence of encapsulated yeast and in Rio Grande do Sul, Abegg et al. [36] isolated *C. neoformans* var. grubii in 87% of Psittacidae excreta samples. The percentage presented in this study, conducted in what is considered a closed environment, is in agreement with the data presented in the study by Kobayashi et al. [34], who demonstrated that the presence of the fungus in environmental samples is more representative when such samples are protected from the weather. In Brazil, no reports concerning the ecology of this species were identified exclusively in the presence of library dust. Isolates from 41 colonies of seven species of *Cryptococcus* spp. identified in the microhabitat of dust shows the importance of knowledge regarding the saprobiotic sources that host the *Cryptococcus* spp yeasts responsible for important and fatal cases of cryptococcal meningitis in immunosuppressed and immunocompetent individuals [40-42].

In addition to public places, enclosed environments may contain a high density of *C. neoformans*, as shown in the study conducted by Criseo et al. [43], who reported 26.6% in bird excreta in pet stores and homes. Swinne et al. [44] isolated *C. neoformans* in domestic dust in Bujumbura (South Africa) in samples collected from homes of patients with cryptococcosis associated with AIDS, making a significant correlation between the existence of pigeons close to the home environment and the probability of contamination of these homes. In a study conducted in different regions of Bangkok, Soogarum et al. [45] demonstrated the presence of *C. neoformans* in 14 samples of pigeon droppings. Hanasha et al. [46] analyzed 509 samples of columbiform droppings collected from several Jordanian cities and reported 336 samples positive for *Cryptococcus* spp. However, these studies did not classify these yeasts at the species level. In addition, Passoni et al. [22] isolated basidiomycetous from captive bird droppings, compared with dust from the inside of the home and the) peridomicile. Of the 79 samples of bird droppings collected by these researchers, 12.7% were positive. The authors concluded that the frequency of positive isolates in homes that maintained captive birds was the main factor responsible for the contamination in these homes. In this study, the percentage of isolates of *Cryptococcus* spp in 84 samples collected with swabs resulted in 18 (21.4%) positive samples (Chart 1).

The environmental source of *C. gattii* is associated with decomposing eucalyptus material, as well as other plant materials in the process of decomposition. Nowadays, this species is being identified on different trees and in different geographical regions of Brazil, and has already been isolated on native and introduced plant specimens, such as false sicklepod (*Senna multijuga*), stinking toe (*Cassia grandis*), Chinese banyan (*Ficus microcarpa*), Cabori (*Miroxilum peruiferum)*, Sibipiruna (*Caesalpinia peltophoroides*), and Oiti (*Moquilea tomentosa)*, revealing other natural habitats for this species [16,17,19,28,30,47,48]. While researching trees in the Brazilian Amazon, Fortes et al. [29] reinforced evidence that *C. gattii* is not associated with one species of tree in particular, but rather to a specific habitat niche formed by the natural decomposition of wood. Recent research has proved this hypothesis, as shown by Randhawa et al. [49], who demonstrated the prevalence of *C. gattii* (24%) and *C. neoformans* (26%) in the soil around the base of certain host trees, indicating that the soil is another important ecological niche for these two species of *Cryptococcus.* More recently, Girish et al. [50] confirmed this hypothesis by isolating *C. neoformans* from environmental samples on 40 selected trees, taking into consideration decomposing wood and bits of bark of live trees in the Guindy National Park in Chennai, in the southern region of India, with the very first isolation of *C. gatti* on a species of jambul (*Syzygium cumini*).

Studies performed in several countries, including samples from plant specimens, have revealed the presence and abundance of *C. gattii* in a variety of native species in Australia [1,13], Mexico [51], Egypt [52], Portugal [15], Argentina [53], Colombia [54,55], India [49,50,56] and Canada [57-59].

Last year, while investigating plant species on a Caribbean island in Puerto Rico, Loperena-Alvares et al. [60] detected the presence of *C. gatti* in lesions on succulent plants of the Cactaceae family (*Cephalocereus royenii)*, a type of cactus that is extremely common in the region. More recently, in Colombia, Firacative et al. [61] processed 3,634 samples from trees surrounding the residences of patients afflicted by cryptococcosis caused by *C. gatti*, isolating a sample of *C. gatti* serotype B, two samples of *C. gatti* serotype C and three samples of *C. neoformans* var. grubii serotype A.

Given these facts, the results obtained in this study suggest that the locations researched may be surrounded by abundant varied tree vegetation (*Mangifera indicaCocothrimax spissaCassia fistulaCaesalpinia peltophoroidesEucalyptus camaldulensis*), including species of *Eucalyptus* spp, in which the main spore is represented by basidiospores present in the flowers of this genus of trees that functions as “host tree” for the fungus through a biotrophic association; thus greatly facilitating the dispersion of these spores into the environments analyzed. This fact can be observed in the work of Mahmoud et al. [52] in Egypt, which revealed the presence of *C. gatti* in samples of flowers of *Eucalyptus camaldulensis*, and in the study performed by Velagapudi et al. [42], which used acquisition by inhalation as the environmental criteria.

The basidiomycete yeast *C. neoformans* is well adapted to bird droppings, particularly that of pigeons. The increasing population of birds is becoming an environmental and public health issue in Brazil and the rest of the world. This columbiform species has a habit of living in groups, building its nests high up on buildings, towers, attics and windowsills, among other locations, while it feeds on grains and remnants of food and garbage in public locations [62]. In this study, a large concentration of pigeons (*Columba livia domestica*) was observed in the urban environments near the locations studied and these birds have access to attics and windowsills that are not sealed. Observation verified that the bird droppings remained on roofs and air-conditioning vents, facilitating the dispersal of dry particles containing fungal spores inside the facilities. In a recent study conducted by Takahara et al. [63], which analyzed samples of pigeon droppings in the city of Cuiabá, state of Mato Grosso, Brazil, collected from several domestic, commercial, and public environments, the presence of strains of *C. neoformans* was demonstrated in bird droppings.

The meteorological factors of each region, such as temperature, humidity and light exposure, influence and can contribute to obtaining different results [40,64,65]. Granados and Castañeda [65] have demonstrated the prevalence of *Cryptococcus* species, suggesting that meteorological conditions influenced the presence of this yeast and that *C. neoformans* is more frequently isolated during wetter seasons on dry droppings. The microclimatic conditions in the city of Cuiabá, Brazil, with an average temperature between 34 and 35°C, a dry winter, and a rainy summer, can favor the development and dispersion of fungi in the environment. The microclimates, (temperature and humidity) identified in these environments are considered to be factors for the presence of these microorganisms in the locations studied. In addition to the relation of these ligninolytic basidiomycetes decomposers of cellulose and other plant materials, the greater frequency of *C. gattii* (15; 36.6%) in relation to *C. neoformans* (3; 7.3%) may be directly influenced by temperature, since according to Ishaq et al. [66], *C. neoformans* does not grow in temperatures above 40°C and is sensitive to direct sunlight. However, in a study performed by Kobayashi et al. [34], the authors affirmed that humidity is another factor that also seems to influence the viability of *C. neoformans* during collection and possibly on the swab samples, that tends to affect bacterial decomposition, thus altering the pH and probably inhibits the proliferation of the yeast.

Cryptococcosis is generally related to infection by *C. neformans* and *C. gattii* and is rarely caused by other species, including *C. albidusC. laurentiiC. curvatus* and *C. uniguttulatus*[23]. According to Khawcharoenporn et al. [67], the other species of the genus *Cryptococcus* are generally considered to be saprophytes. However, in the last few decades, reports have appeared in the literature regarding infections caused by other species, with *C. albidus* and *C. laurentii* being responsible for 80% of the cases of cryptococcosis caused by organisms other than *C. neoformans* and *C. gattii*. With the increase in the number of patients presenting compromised immune systems and the ample use of immunosuppressant agents, the incidence of fungal infections has increased worldwide, including those caused by emerging *Cryptococcus* species such as *C. albidus* and *C. laurentii *[68]. In this study, the isolation of 15 (36.6%) *C. gattii* samples; 3 (7.3%) *C. neoformans* samples, and 2 (4.9%) *C. albidus* samples (Table 1) demonstrated that the presence of species considered to be potentially pathogenic, as well as emerging species, may reflect the possibility of such species, which are present in a wide variety of substrates, constituting infectious agents for cryptococcosis.

The results obtained in this study indicate evidence of the diversity of the environmental origin of *Cryptococcus* spp and highlights the substrates favorable to their respective development in the environment. The microbiota analysis of dust from locations where human presence is constant is necessary to implement health surveillance programs to protect the health of workers, as well as preventing possible sources of acquisition of infectious disease-causing organisms, including cryptococcosis.

## Abbreviations

KOH, Potassium hydroxide; AFLP, (*Amplified fragment length polymorphism*); CGB, Medium (L-canavanine glycine bromothymol blue).

## Competing interests

The authors declare that they have no competing interests.

## Authors' contributions

All authors collected the biological material. DPLJ designed the study, performed and was involved in drafting the manuscript. All authors were involved performed in examination of biological materials, in data analysis and drafting of the papers. RCH participated in revising the manuscript and was the researcher in chief. All authors have read and approved the final manuscript.
